# Evaluation of serum zonulin for use as an early predictor for gestational diabetes

**DOI:** 10.1038/nutd.2017.9

**Published:** 2017-03-20

**Authors:** K Mokkala, K Tertti, T Rönnemaa, T Vahlberg, K Laitinen

**Affiliations:** 1 Institute of Biomedicine, University of Turku, Turku, Finland; 2 Functional Foods Forum, University of Turku, Turku, Finland; 3 Department of Obstetrics and Gynecology, Turku University Hospital and University of Turku, Turku, Finland; 4 Department of Medicine, Institute of Clinical Medicine, University of Turku and Turku University Hospital, Turku, Finland; 5 Department of Clinical Medicine, Biostatistics, University of Turku and Turku University Hospital, Turku, Finland

## Abstract

Diet has an important role in regulating intestinal permeability and subsequently the risk for metabolic disorders. In this observational study, we examined whether serum intestinal permeability marker zonulin, could be used as a predictor for gestational diabetes mellitus (GDM). Serum zonulin concentration was measured in early pregnancy in overweight or obese pregnant women (*n*=88) at risk for developing GDM. Serum zonulin was associated with higher odds of GDM (adjusted OR for 1 ng ml^−1^ increase in zonulin: 1.08, 95% CI: 1.02–1.15; *P*=0.009), diagnosed by a 2-h 75-g oral glucose tolerance test at late pregnancy. The optimal cutoff value was 43.3 ng ml^−1^, with sensitivity of 88% (95% CI: 71–100%) and specificity of 47% (95% CI: 33–58%). The area under the ROC-curve was 0.67 (95% CI: 0.54–0.81). Our results show an association between increased early-pregnancy serum zonulin concentration and GDM, suggesting zonulin as a possible predictor for GDM.

## Introduction

Diet and lifestyle, particularly obesity, are well-established risk factors for gestational diabetes (GDM). Zonulin is a proposed serum marker for intestinal permeability,^1^ the increased concentrations reflecting an increased intestinal permeability. This, in turn, has been shown to positively associate with elevated inflammatory markers^2^ and again with insulin resistance^3, 4^ in non-pregnant populations. Diet composition has recently been shown to associate with serum zonulin concentration,^5^ suggesting a new mechanism for how diet may contribute to the onset of inflammation associated metabolic disorders.

We investigated whether an increased serum zonulin concentration could be used as a tool to predict GDM in early pregnancy, as reliable markers are lacking. Currently, the diagnosis of GDM is based on the oral glucose tolerance test (OGTT) generally performed at the second trimester of the pregnancy.

## Research design and methods

This pilot study comprised overweight or obese women at risk for GDM from an ongoing mother-infant dietary intervention trial aimed at preventing GDM (ClinicalTrials.gov, NCT01922791). This study was conducted according to the guidelines laid down in the Declaration of Helsinki and all procedures involving human subjects were approved by the Ethics Committee of the Hospital District of Southwest Finland (permission number 115/180/2012). Written informed consent was obtained from all subjects. Hundred women at early pregnancy (the baseline study visit; mean 12.8, s.d: 2.5 weeks of gestation), were included in this study for analysis of serum zonulin using a Zonulin ELISA kit (Immundiagnostik AG, Bernsheim, Germany; inter-assay variation 10.6%).

GDM was diagnosed based on 2-h 75-g OGTT if one or more values were above the threshold level: 0 h ⩾5.3, 1 h ⩾10.0, 2 h ⩾8.6 mmol l^−1^ (The Finnish Medical Society guidelines, which are in accordance with the American Diabetes Association 2007 guidelines). Twelve subjects either withdraw from the study (*n*=9), had miscarriage (*n*=2) or refused the OGTT (*n*=1), and thus, the final study population comprised 88 women. BMI of the women did not differ between those who remained in the study (mean 30.8, s.d.: 4.5) compared to those who dropped out (mean 30.0, s.d.: 5.3).

The OGTT was performed at 26 (s.d.: 3) weeks of gestation (midpregnancy OGTT). In high-risk mothers (*n*=25) the OGTT was performed already at 14 (s.d.: 2) weeks of gestation (early-pregnancy OGTT) and if negative retest was performed at midpregnancy.

### Statistics

Serum zonulin concentrations were compared between mothers with or without GDM by independent-samples *t*-test. The association between serum zonulin and GDM was analyzed with binary logistic regression after adjustment for BMI, previous GDM (*n*=5), diagnosis of type 2 diabetes or metabolic syndrome of the parents of the mother and study group of the original intervention setting. Receiver-operating characteristics (ROC) analysis and the Youden index (sensitivity+specificity-1) were calculated to find the optimal cutoff value for serum zonulin to predict GDM. The maximum value of the Youden's index was used as a criterion for selecting the optimal cutoff value for serum zonulin. Sensitivity, specificity, positive predictive value (PPV) and negative predictive value (NPV) were determined. Statistical analyses were performed with IBM SPSS Statistics for Windows version 23.0 by a statistician (TV) independent of the study planning and execution, and other researchers were kept blind with regard to the study code.

## Results

The mean (s.d.) age of the women was 30.1 (4.9) years and prepregnancy body mass index (BMI) was 30.8 (4.3) kg m^−2^. Forty-three percent were overweight (BMI 25–29.99 kg m^−^^2^), 57% were obese (BMI⩾30 kg m^−2^) and 42% were primiparas. GDM was diagnosed in 24 mothers out of 88 (27%). Eight were diagnosed in early pregnancy, and 16 were diagnosed in midpregnancy. No statistically significant differences in baseline parameters were detected between the women with GDM compared to those remaining without GDM (Table 1).

**Figure 1:**
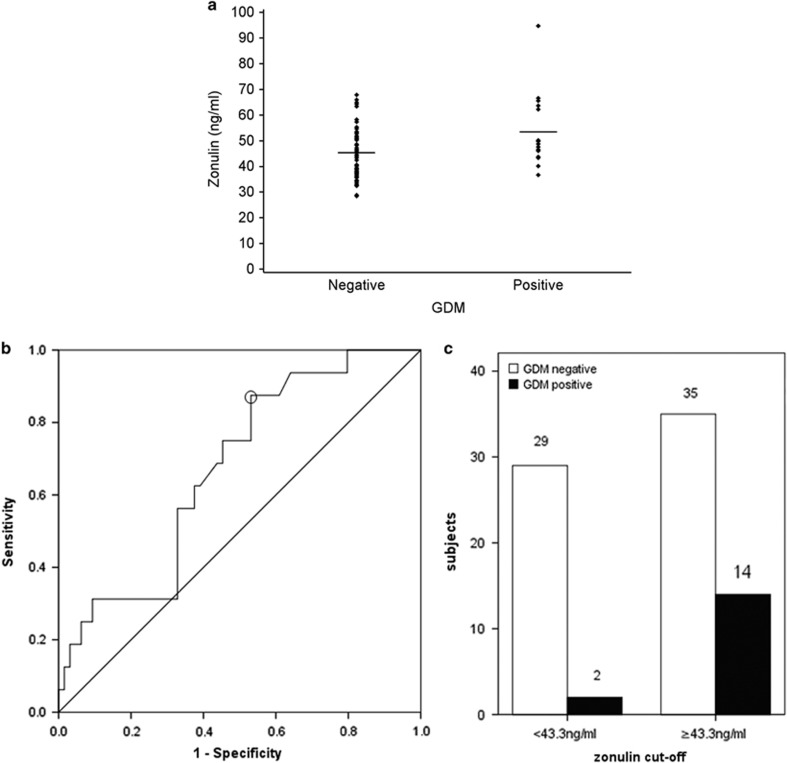
(**a**) Dot blot of early-pregnancy serum zonulin values according to GDM positive and negative subjects at midpregnancy. (**b**) ROC curve for serum zonulin concentration to predict the GDM. AUC was 0.67 (95% CI: 0.54 to 0.81). An optimal cutoff value (sphere in figure) for serum zonulin maximizing the Youden Index was ⩾43.3 ng ml^−1^, with a sensitivity of 88% (95% CI: 71–100%), specificity of 47% (95% CI: 33–58%), positive predictive value 29% (95% CI: 16–41%) and negative predictive value 94% (95% CI: 85–100%). (**c**) Number of GDM positive and negative subjects at midpregnancy according to serum zonulin cutoff (⩾43.3 and <43.3 ng ml^−1^).

The mean (s.d.) serum zonulin concentration was 47.0 (11.2) ng ml^−1^. The early-pregnancy serum zonulin concentration was higher in women who developed GDM at midpregnancy (mean (s.d.) 53.4 (14.3) ng ml^−1^; *n*=16) compared to those who did not (45.2 (9.7) ng ml^−1^, *P*=0.008, *n*=64; Figure 1a). The result was similar when all the women diagnosed positive for GDM were taken into account (serum zonulin in women with 51.6 (13.5) ng ml^−1^, *n*=24 and remaining without GDM 45.2 (9.7) ng ml^−1^, *n*=64, *P*=0.008).

Serum zonulin concentration was associated with higher odds of GDM at midpregnancy (adjusted (for BMI, previous GDM, original intervention group) OR for one-unit (ng ml^−1^) increase in zonulin: 1.08, 95% CI: 1.02–1.15; *P*=0.009). The result was similar when the eight women diagnosed in early pregnancy were included in the analysis (adjusted OR: 1.07, 95% CI: 1.01–1.12, *P*=0.016). After adjustment for type 2 diabetes or metabolic syndrome of the parents of the mother, the results did not change. Using ROC curve analyses (Figure 1b) and the Youden Index, the optimal cutoff value for serum zonulin was ⩾43.3 ng ml^−1^ in predicting midpregnancy GDM, with a sensitivity of 88% (95% CI: 71–100) and specificity of 47% (95% CI: 33–58). This concentration had a PPV of 29% (95% CI: 16–41) and NPV of 94% (95% CI: 85–100). The area under the ROC- curve (AUC) was 0.67 (95% CI: 0.54–0.81; Figure 1b). Further, using the cutoff value, 14 of the GDM positive subjects in midpregnancy had serum zonulin concentration above the zonulin cutoff and two below (Figure 1c).

## Discussion

We demonstrated that serum zonulin concentration measured in early pregnancy was higher in women diagnosed with GDM compared to those remaining without GDM. Importantly, the elevated serum zonulin concentrations were detected prior to GDM diagnosis. Thus, measurement of serum zonulin concentration may be taken as a predictor for an increased risk of GDM, the likely mechanism being induction of inflammation and interference of the action of insulin receptors.^6^

Previously, some other serum markers determined in early pregnancy have been evaluated for use in predicting GDM with a variable success. Measurement of fasting plasma glucose yielded a sensitivity of 47 and specificity of 77%.^7^ Further, HbA1c demonstrated a sensitivity of 19 and specificity of 95%, and fructosamine a sensitivity of 12 and specificity of 95%,^8^ and hs-CRP a sensitivity of 37 and specificity of 87%.^9^ In light of these previous studies, and also a meta-analysis^10^ regarding adiponectin measurement at the first trimester of pregnancy (pooled sensitivity of 60 and specificity of 81%, AUC 0.79), the sensitivity of 88%, in the present study is encouraging. In this study, twelve of the hundred women measured for serum zonulin concentration dropped out. We consider there is no bias due to these drop outs, since BMI did not differ between those who remained in the study compared to withdrawn. This is a pilot study and further research, both with higher number of subjects being in risk group for GDM and with unselected pregnant women, is needed before applying the measurement of serum zonulin for prediction purposes of GDM. It is of note that, currently, the prevalence of overweight is ~30% in young women at fertility age and thus a relatively large proportion of pregnant women are at an increased risk for GDM. Novel markers for identification of subjects that would likely benefit from lifestyle counselling is of importance as encouraging results in reducing the risk for GDM has been recently provided. However, it may also be reasonable to only test women being in high-risk group for GDM, as this would result in effective screening for those in need for intensified lifestyle modification already at early pregnancy. Further, the measurement result itself may act as a motivator for lifestyle modifications. The suboptimal level of specificity detected (47%) in zonulin measurement is thus of less importance. However, the possibility of false positivity needs to be taken into account in counselling. We would like to emphasize the importance of individual counselling in which the known risk factors for the onset of GDM are taken into account as a whole.

It is proposed here that the serum zonulin concentration, measured from a single blood sample, may be used as a marker for glucose metabolism, particularly in large population based dietary studies.

## Figures and Tables

**Table 1:** Clinical characteristics of the women remaining healthy and developing GDM in early pregnancy

	*Women remaining without GDM (*n=*64)*	*Women developing GDM (*n=*16)*
Gestational weeks (mean (s.d.))	12.9 (2.5)	12.9 (2.1)
BMI (mean (s.d.))	30.4 (4.4)	30.9 (3.2)
Prepregnancy BMI (mean (s.d.))	30.1 (4.4)	30.1 (3.5)
Age (mean (s.d.))	30.1 (4.8)	29.5 (3.7)
Primipara (*n* (% of subjects in group))	26 (41%)	7 (44%)
Previous GDM (*n* (% subjects in group)	4 (6.3%)	1 (6.3%)
Gestational weeks at the time of GDM diagnosis	25.6 (2.2)	25.6 (2.7)
